# Supplementary figures and images for: Novel Cluster AZ Arthrobacter phages Powerpuff, Lego, and YesChef exhibit close functional relationships with Microbacterium phages
Source: PLoS One. 2022 Jan 13;17(1):e0262556. doi: 10.1371/journal.pone.0262556 (PMC8758107; doi:10.1371/journal.pone.0262556)

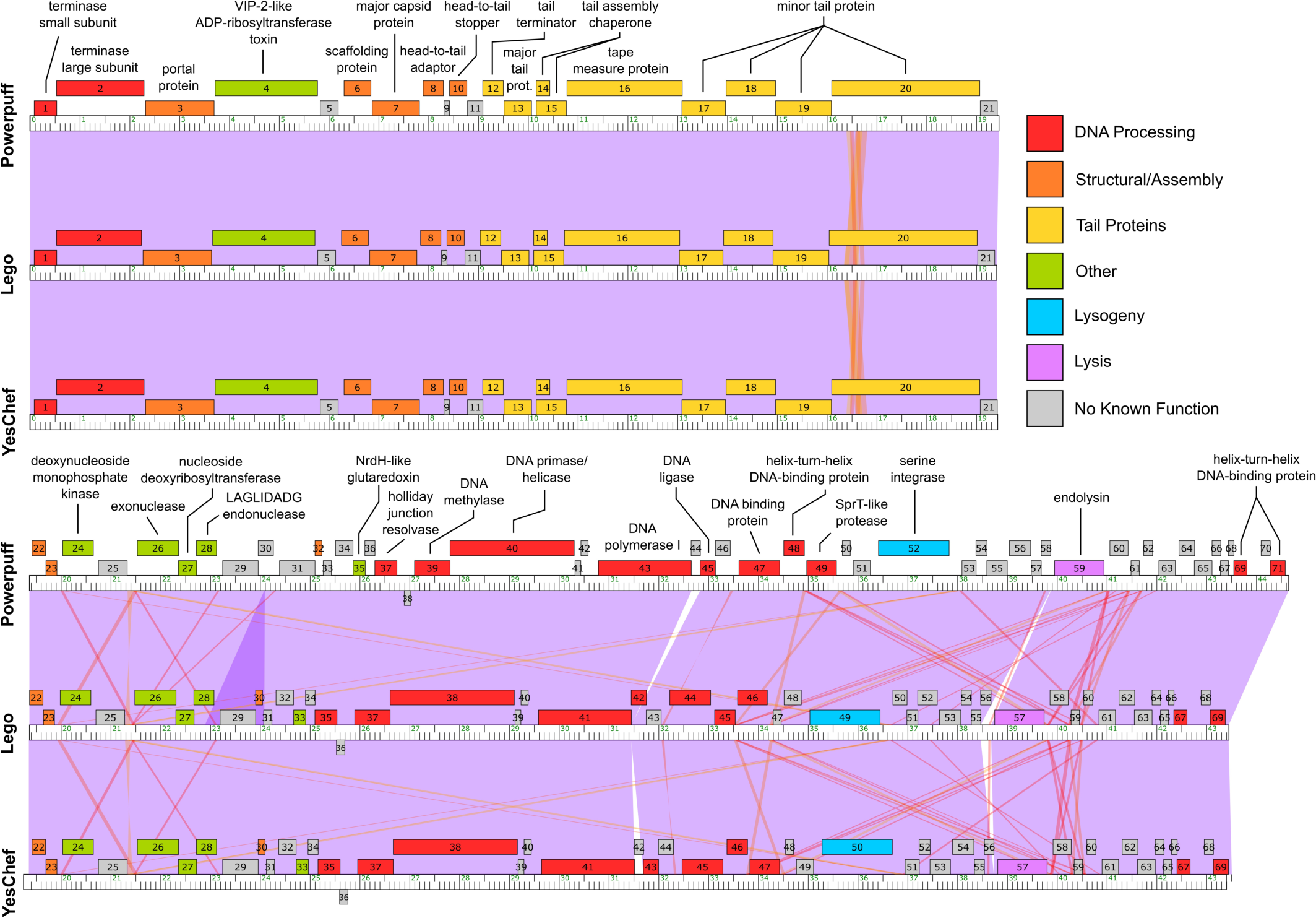

Supplement: S1 Fig — Genomes were downloaded from Phamerator and formatted using Inkscape 1.0. Genes were sorted by general function or type, as indicated in the legend above. Powerpuff, Lego, and YesChef have highly similar genomes, with pairwise BLASTn scores of over 98.63% identity with at least 99% coverage and E-values of 0. Each genome was found to be between 43,446 and 44,651 bp in length, encoding between 69 and 71 genes. Notable dissimilarities included a gene duplication in phage Powerpuff (Powerpuff_29 and Powerpuff_31), which twice encoded a gene of unknown function that was present only once in phages Lego and YesChef. Phage Lego was found to encode a gene of unknown function (Lego_56) not found in Powerpuff or YesChef, located directly upstream of the gene encoding an endolysin. Phages Powerpuff and YesChef also encoded a gene of unknown function (Powerpuff_44 and YesChef_42) not found in phage Lego. This gene was positioned within a cassette of DNA processing genes in these phages. (TIF) [file pone.0262556.s001.tif]
