# Supplementary material for: Novel Cluster AZ Arthrobacter phages Powerpuff, Lego, and YesChef exhibit close functional relationships with Microbacterium phages
Source: PLoS One. 2022 Jan 13;17(1):e0262556. doi: 10.1371/journal.pone.0262556 (PMC8758107; doi:10.1371/journal.pone.0262556)
Supplement: Attachment — Submitted filename: Response to Reviewers_Kapinos.docx [file pone.0262556.s007.docx]

**Journal Requirements**

Style guidelines were checked and revised as necessary. References were reviewed and no retracted articles where identified.

**Reviewer #1:**

*Introduction:*

*Line 88 to 110 describe the finds of this study. It doesn’t make any sense to write findings/results under introduction. Take these out from introduction, instead, please state clearly the aim of this study here.*

Thank you for the feedback, we have revised the last paragraph of the introduction to state the goals of the paper more clearly. In constructing this paper, we followed the structure described in Mensh B, Kording K (2017) “Ten simple rules for structuring papers,” PLOS Computational Biology 13(9): e1005619. <https://doi.org/10.1371/journal.pcbi.1005619>. This editorial recommends that the summary of the introduction should include the approach and brief results. We have however significantly condensed this section.

*Methodology:*

*Line 163. Please expand the host range assay.*

The host range assay methodology has been expanded with additional detail.

*Results:*

*Line 267, please define what does this “weak alignment” refers here??*

Section has been revised.

*Discussion:*

*Line 441. At the end please add examples of those few other host genera??*

*Add the weakness of this study.*

Line clarified to reflect that we are referring to actinobacterial genera. Line added on the weakness of comparative genomics studies being that clustering parameters rely on limited available data. Line added acknowledging lifecycle conclusions were based on wetlab data from a single phage.

**Reviewer #2:**

*Minor corrections and suggestions:*

*The writing and language usage is correct and orthodox, however, there are many instances where reader gets breathless. There are many instances where a colon or a semicolon can be the place to cut a sentence (i.g. semicolon at L37P2 in the abstract section). Indeed, it is easy to find sentences spanning 7-10 lines. This reviewer thing it is necessary to cut the length of several sentences.*

Thank you for the constructive feedback. The manuscript has been edited throughout to reduce verbosity and improve clarity.

*The complexity of presenting scientific data is more of a problem that a virtue. The manuscript quickly run into the topic of bacteriophage clusters with designed names that will be unfamiliar, unless reader is really into the Arthrobacter world and Arthrobacter phages. To easy the understanding, and because there are few restrictions in the number of figures and tables, this reviewer suggests a simple table with the names of phage clusters, the hosts, number of phages and any relevant information (presence of lytic, temperate phages may be informative).*

A supplemental table (S4 Table) has been added with summary information of actinobacteriophage cluster data.

**Reviewer #4:**

*In Fig. 7; It is suggested to put the bootstrap value(s) to closely perceive the intra-cluster similarities in that phylogenic tree.*

SplitsTree network phylogenies are different from phylogenetic trees in that they typically do not have bootstrap values. In a classic phylogenetic tree, relationships are predicted with a given confidence value based on sequence (dis)similarities. With a network phylogeny, SplitsTree uses the presence or absence of each gene to construct a "network" that represents such differences visually. This is less prediction-based, and thus confidence values are not required (for instance, works by other phage scientists do not present bootstrap values on these types of trees). Lines were added to the comparative genomic analysis section of the methods and the results section for figure 7 to address the potential confusion.

*In Fig.8. Plate B; Powerpuff lysogen titer spot was identified with weak signal (no. 7-9). But it was not discussed well either those are individual copies or mutant of Powerpuff phages.*

In this experiment, spot tittering was performed to get a rough estimate of the number of phages released from the lysogen cells. For each spot the presence of clearings indicates phage in that dilution and a rough titer can be calculated since we see two individual plaques at 1e-9. We have no way of determining if released phages are mutants from this assay. The methods section and figure legend for this experiment have been revised for clarity.
